# Characteristics of refractory disease and persistent symptoms in inflammatory arthritis: Qualitative framework analysis of interviews with patients and health care professionals

**DOI:** 10.1111/bjhp.12780

**Published:** 2025-01-08

**Authors:** Hema Chaplin, Carol Simpson, Kate Wilkins, Jessica Meehan, Nora Ng, James Galloway, Ian C. Scott, Debajit Sen, Rachel Tattersall, Rona Moss‐Morris, Heidi Lempp, Sam Norton

**Affiliations:** ^1^ Health Psychology Section Institute of Psychiatry, Psychology and Neuroscience, King's College London London UK; ^2^ Centre for Rheumatic Diseases, Department of Inflammation Biology King's College London London UK; ^3^ Guy's and St Thomas' NHS Foundation Trust London UK; ^4^ King's College Hospital NHS Foundation Trust London UK; ^5^ Primary Care Centre Versus Arthritis, School of Medicine Keele University Keele UK; ^6^ Haywood Academic Rheumatology Centre, Haywood Hospital, Midlands Partnership University NHS Foundation Trust Stoke‐on‐Trent UK; ^7^ University College London Hospitals NHS Foundation Trust London UK; ^8^ Versus Arthritis Centre for Adolescent Rheumatology University College London London UK; ^9^ Sheffield Teaching Hospitals NHS Foundation Trust Sheffield UK; ^10^ Barbara Ansell National Network for Adolescent and Young Adult Rheumatology UK

**Keywords:** inflammatory arthritis, multi‐disciplinary health care professionals, persistent symptoms, qualitative, refractory disease

## Abstract

**Objectives:**

This study aims to explore patients' and clinicians' understanding and experiences of refractory disease (RD) and persistent physical and emotional symptoms (PPES) in patients with inflammatory arthritis (IA), namely rheumatoid arthritis or polyarticular juvenile idiopathic arthritis from their perspectives through interviews and/or focus groups.

**Design:**

A qualitative study was conducted, following a pragmatic epistemology approach with framework analysis employed.

**Methods:**

Semi‐structured interviews or focus groups with IA patients (*n* = 25) and multi‐disciplinary rheumatology HCPs (*n* = 32) were conducted at one time point to obtain participants respective understanding and experiences of managing RD/PPES, and its impact on the patient‐professional relationship.

**Results:**

Three key themes were identified from both patients and professionals' experiences of RD/PPES: (1) relevant treatment experiences, (2) symptoms (with or without inflammation) and (3) impact: physical, psychological and social. These themes included 28 specific categories that would be considered as components characterizing RD/PPES, most common to both patients and HCPs with six being patient‐specific and only one HCP‐specific. The specific biopsychosocial symptoms and impacts of RD/PPES pertain to pain, fatigue, stiffness, joint involvement and physical, psychological and social functioning and quality of life, covering disease‐related distress, mobility and independence. Wider influential factors such as comorbidities, non‐adherence, health/medication beliefs and behaviours and social support were also identified.

**Conclusion:**

Common persistent symptoms that have both mental and physical impact characterize RD/PPES in IA and therefore a more integrated holistic approach to treatment is needed from multi‐disciplinary HCPs, including health psychologists.

**Statement of Contribution**

**What is already known on this subject?**

- Various definitions for refractory disease (not responding to treatment) have been proposed for rheumatoid arthritis (Chaplin et al., 2021).
- These do not consider perspectives of patients, those with juvenile‐onset disease or persistent symptoms, or multi‐disciplinary health care professionals.

**What does this study add?**

- This is the first study to qualitatively explore refractory disease and persistent symptoms in inflammatory arthritis from both patient and clinician perspectives.
- RD/PPES are persistent symptoms with biopsychosocial impact, either in the presence or absence of inflammation despite treatment with multiple drugs.
- Specific biopsychosocial symptoms and impact (e.g. disease‐related distress and physical functioning) were identified as well as wider influential factors (e.g. health‐related behaviours and beliefs) that could be targeted through interventions from multi‐disciplinary HCPs.

## INTRODUCTION

Inflammatory arthritis affects ~3% of the global population (Hoving et al., 2014), causes joint pain, stiffness and swelling due to inflammation and are associated with reduced quality‐of‐life (Matcham et al., 2014; National Audit Office, 2009). Rheumatoid arthritis (RA), accounts for three‐in‐five cases of inflammatory arthritis, which is an adult‐onset condition. Those under the age of 16 are diagnosed with the juvenile‐onset equivalent polyarticular juvenile idiopathic arthritis (PolyJIA) with similar disease presentation and prognosis to RA (Wallin et al., 2009). Controlling inflammation remains the primary goal of treatment (Silman & Pearson, 2002) to reduce joint damage and permanent function loss, with secondary goals to improve pain and joint stiffness/mobility. The current Inflammatory Arthritis treatment paradigm is treat‐to‐target, with remission or low disease activity as the target (Ravelli et al., 2018; Smolen et al., 2010). This strategy is implemented through early intensive treatment using conventional disease‐modifying anti‐rheumatic drugs (DMARDs), with add‐on biologic therapy (NICE, 2009, 2015).

Patients with inflammatory arthritis not achieving this low disease activity target by not responding to treatment were previously considered to have refractory disease (RD) (Chaplin et al., 2021; Polido‐Pereira et al., 2011). Recent research initiatives have focused on redefining this concept primarily in RA (Buch et al., 2021; Nagy et al., 2020). However, these definitions do not account for patients' perspectives, or those with persistent emotional and physical symptomology (PPES) such as pain and fatigue, despite well‐controlled inflammation as other targets to be achieved (Schoemaker & de Wit, 2021; Stevenson et al., 2016). Additionally, the perceived impact for those with juvenile‐onset disease is not considered, such as PolyJIA (Chaplin et al., 2023). The difference between patients' and clinicians' definitions of RD in inflammatory arthritis and its implications has been identified as a key knowledge gap (Young, 2015).

Incorporating the patient perspectives into the RA remission definition has been explored qualitatively (van Tuyl et al., 2015). Given RD is the opposite end of the disease activity‐treatment experience spectrum to remission, incorporating patient perspectives about their experiences of RD/PPES as well as health care professional perspectives would equally be meaningful. The evidence base is focused on RA but there is clear justification for investigating PolyJIA by taking a transdiagnostic approach as PPES are poorly understood but common across ages and conditions. The evidence gap and importance of research into refractory PolyJIA in adults has also been highlighted nationally (BANNAR, 2017), hence their inclusion in this study. Most work in this area has been conducted by rheumatologists with a narrow focus on inflammation which may not take other perspectives into account to understand what, why and how symptoms (PPES) or disease (RD) persist despite improved treatment.

The symptom severity and impact on people's lives that arise from a long‐term condition cannot be purely explained from a biomedical viewpoint (Picariello et al., 2023). Therefore to fully explore wider biopsychosocial explanatory elements, cognitive‐behavioural processes as detailed by the self‐regulation theory (Leventhal et al., 2016) and necessity‐concerns (Horne et al., 2019) for illness/treatment representations were considered to understand the relevant cognitions, behaviours and emotions in RD/PPES. Additionally, the Adjustment model (Carroll et al., 2022; Moss‐Morris, 2013) may also help to understand why those with RA/PolyJIA progress to experience RD/PPES due to the non‐static nature of the illness experience. Using health psychology models and frameworks is important to fully understand a concept and consider mechanisms involved for understanding complex constructs (Rimer & Glanz, 2005), which is currently missing for characterizing RD/PPES. There is an absence of a systematic approach to identify, conceptualize or evaluate RD (Buch, 2018), with a clear gap regarding what health care professionals and people living with RA/PolyJIA understand about RD/PPES. To our knowledge there have not been any qualitative studies published in this area that have been conceived using health psychology theory. Given this gap in the literature and the complex nature of RD/PPES across RA and PolyJIA, this qualitative exploration sought to capture a range of experiences and beliefs in patients with RA and PolyJIA, and multi‐disciplinary health care professionals involved in their care. This study aims to explore patients' and clinicians' understanding and experiences of RD/PPES in Inflammatory Arthritis.

## METHODS

### Design and sample

A qualitative study design was conducted with: (a) one‐to‐one patient interviews and (b) interviews and focus groups with multi‐disciplinary rheumatology health care professionals. This methodology allowed systematic examination of the experiences and understanding of RD/PPES in RA and PolyJIA from the perspectives of both recipients and providers of care. A pragmatic epistemology approach was followed (Johnson & Onwuegbuzie, 2004), aligning with framework analysis (Ritchie & Spencer, 1994) (see Appendix S6 for additional methodological information). Framework analysis enables comparisons between and within participants to be made on data collected from individual interviews and focus groups to capture similarities and differences (Ritchie & Spencer, 1994; Srivastava & Thomson, 2009). Both patients and health care professionals were recruited from outpatient rheumatology clinics in one Midlands and three London NHS Trusts and from a national network of rheumatology HCPs in England. Full NHS ethical approval granted by London—Hampstead Research Ethics Committee (18/LO/1171, June 2018).

Patients were purposively recruited (Palinkas et al., 2015), stratified during screening by disease type, gender and inflammation (low (PPES) or high (RD)) to ensure appropriate participant representation in line with the research question and sufficient information power (Malterud et al., 2016). At each site, health care professionals were identified and approached by the principal investigator. A list of interested staff was provided to the researcher to then contact for recruitment. Detailed inclusion/exclusion criteria for the study are detailed in Table 1. Eligible patients were: (a) those aged ≥16 years, (b) who had PolyJIA or RA for >2 years, (c) on their third treatment and (d) with RD/PPES as determined by the DAS28/JADAS10 or Patient Global Assessment (Consolaro et al., 2014; Fransen et al., 2003; Nikiphorou et al., 2016; Wells et al., 2005). Those meeting this criteria were approached at routine clinical appointments. Eligible health care professionals were those currently working in Rheumatology for >1 year across recruiting sites. All participants provided written informed consent before participation and were told the study purpose as portrayed here to explore their experiences of persistent symptoms. To maintain anonymity of contributors, recruitment sites are reported as centres A–E and only aggregate data are presented (Morse & Coulehan, 2015).

**TABLE 1:** Inclusion and exclusion criteria.

Patients	
Inclusion criteria	Exclusion criteria
Clinical diagnosis of PolyJIA or RA Established disease with a duration longer than 2 years Aged 16 years old and above Patients under the care of a rheumatologist at recruiting sites attending outpatient rheumatology clinics RD: Moderately active disease as defined by either DAS28 > 3.2 for RA (Fransen et al., 2003), or JADAS10 > 3.8 for PolyJIA (Consolaro et al., 2014) where used PPES: if Patient Global Assessment score ≥5/10 (Nikiphorou et al., 2016; Wells et al., 2005) and DAS28 > 2.6 or JADAS10 > 1.0 in the last 3 to 6 months Previously did not respond to two conventional/synthetic disease‐modifying anti‐rheumatic drugs (DMARDs) and currently on one biologic DMARD Experiencing persistent physical and emotional symptoms such as pain and fatigue, lasting for at least 3 months and affecting functioning	Diagnosis of other rheumatic disease (e.g. other subtypes of JIA or osteoarthritis) Early disease with duration less than 2 years Under 16 years of age Severe comorbidities such as mental health or respiratory issues, for example, schizophrenia, severe depression and pneumonia Participants unable to give written informed consent, complete questionnaires or participate effectively in the interview due to: Insufficient command of English Significant learning disability Reduced cognitive capacity

Health care professionals	
Inclusion criteria	Exclusion criteria
Health care professionals (e.g. consultants and nurses and allied health professionals) who are currently working in rheumatology across recruiting sites and have worked in rheumatology for >1 year.	Unqualified health care professionals who are currently working in rheumatology across the study sites or have worked in rheumatology for <1 year.

Abbreviations: DAS28, Disease Activity Score‐28 joints, DMARD, disease‐modifying anti‐rheumatic drug, JADAS10, Juvenile Arthritis Disease Activity Score‐10 joints, PolyJIA, polyarticular juvenile idiopathic arthritis, PPES, persistent physical and emotional symptoms, RA, rheumatoid arthritis, RD, refractory disease.

### Data collection, analysis and methodological integrity

The audio‐recorded data were gathered by HC (female PhD student) through face‐to‐face or telephone interviews/focus groups at one time point, using semi‐structured interview guides with open questions that were pilot‐tested (Britten, 1995) (see Appendix S1). The interview schedules were based on relevant literature (Lempp et al., 2012; Minnock et al., 2017; van Tuyl et al., 2008) and the researchers' experiential knowledge, with refinements suggested by patient research partners (CS/KW). The schedules also covered specific categories (e.g. physical/psychosocial impairments and contributing factors) to enable implementation of biopsychosocial cognitive‐behavioural theory into practice (Jones et al., 2002).

The following three key areas explored patient experiences of RD/PPES: (1) experiences of illness and medications, (2) development of illness, symptoms and its impact and (3) expectations and experiences of management. The following areas were explored in HCP focus groups and interviews: (1) experiences of managing patients with RD: (a) symptom presentation, (b) disease progression, (c) language used and descriptions and (d) clinical management and (2) impact on the HCP–patient relationship. Interviews/focus groups took place between 2 November 2018 and 16 January 2019. Interview length ranged from 21 to 51 min, with focus groups ranging from 64 to 78 min. All focus groups were conducted in person (*n* = 20), with four interviews conducted face‐to‐face and eight over the telephone. Face‐to‐face interviews were held in private rooms in the HCPs' clinical or academic setting depending on health care professional preference, or at site E a bi‐annual meeting (*n* = 10). Telephone interviews were conducted in a private university room in London (*n* = 8).

HC had relevant qualitative training and research experience in conducting interviews and focus groups. Patients completed sociodemographic and musculoskeletal health‐related quality‐of‐life questionnaires once (Hill et al., 2016). Health care professionals completed one project‐specific demographic questionnaire. Descriptive statistics such as frequencies or means/medians as appropriate were reported. Study identification numbers were used to ensure anonymity and other potentially identifying data was removed.

The interview transcripts were subsequently imported into NVivo 12 (QSR, 2012). Reporting follows the APA Journal Article Reporting Standards for Primary Qualitative Research (Levitt et al., 2018) to ensure transparency and quality of reporting (Shaw et al., 2019). At various stages (See Appendix S6), three coders (HC, JM and HL) discussed codes/subthemes to assure consistency and credibility across themes and interpretations. Framework analysis was chosen to outline a comprehensive review of collected narratives, driven by participants' original accounts and provided an in‐depth systematic analysis between and within individual experiences of patient and multi‐disciplinary health care professional data (Gale et al., 2013; Jones et al., 2021). Framework analysis is conducted through five steps (Leal et al., 2015; Ritchie & Spencer, 1994): (i) familiarization with data, (ii) preliminary inductive thematic analysis of the whole dataset to develop initial themes, (iii) application of themes again to the whole dataset systematically, (iv) reducing data from transcripts into summaries and organizing these in a matrix (participants by themes) and (v) identifying patterns and relationships across participants and themes.

## RESULTS

Of the 60 eligible patients approached, 26 consented and 25 completed interviews (see Appendix S3; one consented patient did not respond to further contact to arrange the interview). Twenty people living with RA (80%) and five adults with PolyJIA (20%) were interviewed, reflecting disease and gender prevalence within these rheumatic conditions (see Table 2), who had either RD (*n* = 21) or PPES (*n* = 4).

**TABLE 2:** Patient sociodemographic and clinical characteristics.

	Aggregate averages (*n* = 25)
Sociodemographic	
Age, *median (IQR)*	59 (32)
Gender	
Female	84%
Male	16%
Ethnicity	
White British	76%
Black/Black British	8%
Asian/Asian British	8%
White Irish	4%
Mixed	4%
Place of birth	84% UK
Had to stop/modify education/employment due to RA/PolyJIA	64%
Registered disabled	64%
MSK‐HQ, *mean (SD)*	
Total score (out of 56)	24.6 (9.89)
Days physically active (out of 7)	1.0 (1.54)
Clinical	
Inflammatory arthritis diagnosis	
Rheumatoid arthritis	20
Polyarticular juvenile idiopathic arthritis	5
Disease duration (years), *median (IQR)*	20 (14)
Age at diagnosis, *median (IQR)*	28 (29)
Disease activity (DAS28 categories [Fransen et al., 2003])	
Remission (DAS28 < 2.6)	0%
Low (DAS28 ≥ 2.6 and ≤3.2)	16%
Moderate (DAS28 > 3.2 and ≤5.1)	60%
High (DAS28 > 5.1)	24%
Time to first DMARD (months), *median (IQR)*	6.75 (14.6)
Previous number of DMARDs experienced, *median (IQR)*	6 (3)

Fifty‐nine health care professionals were approached, 33 consented and 32 took part in this study (see Appendix S3; one consented professional was not interviewed as felt that their role was well represented during data collection). Five focus groups (*n* = 20) and 12 individual interviews were conducted from 11 hospital trusts (71.9% female), with 7 of these settings outside of London. Professionals represented rheumatology (consultant (*n* = 15) and specialist registrar (*n* = 4)), clinical specialist nursing (*n* = 4), psychology (*n* = 2) and physiotherapy (*n* = 2), occupational therapy (*n* = 2), podiatry (*n* = 1), pharmacy (*n* = 1) and social work (*n* = 1). The mean years of rheumatology experience was 11.72 (SD 7.14) demonstrating this sample is quite experienced. The majority were trained in adult medicine (71.9%) and had received specific musculoskeletal training (84.4%).

### Themes characterizing RD/PPES

**FIGURE 1:**
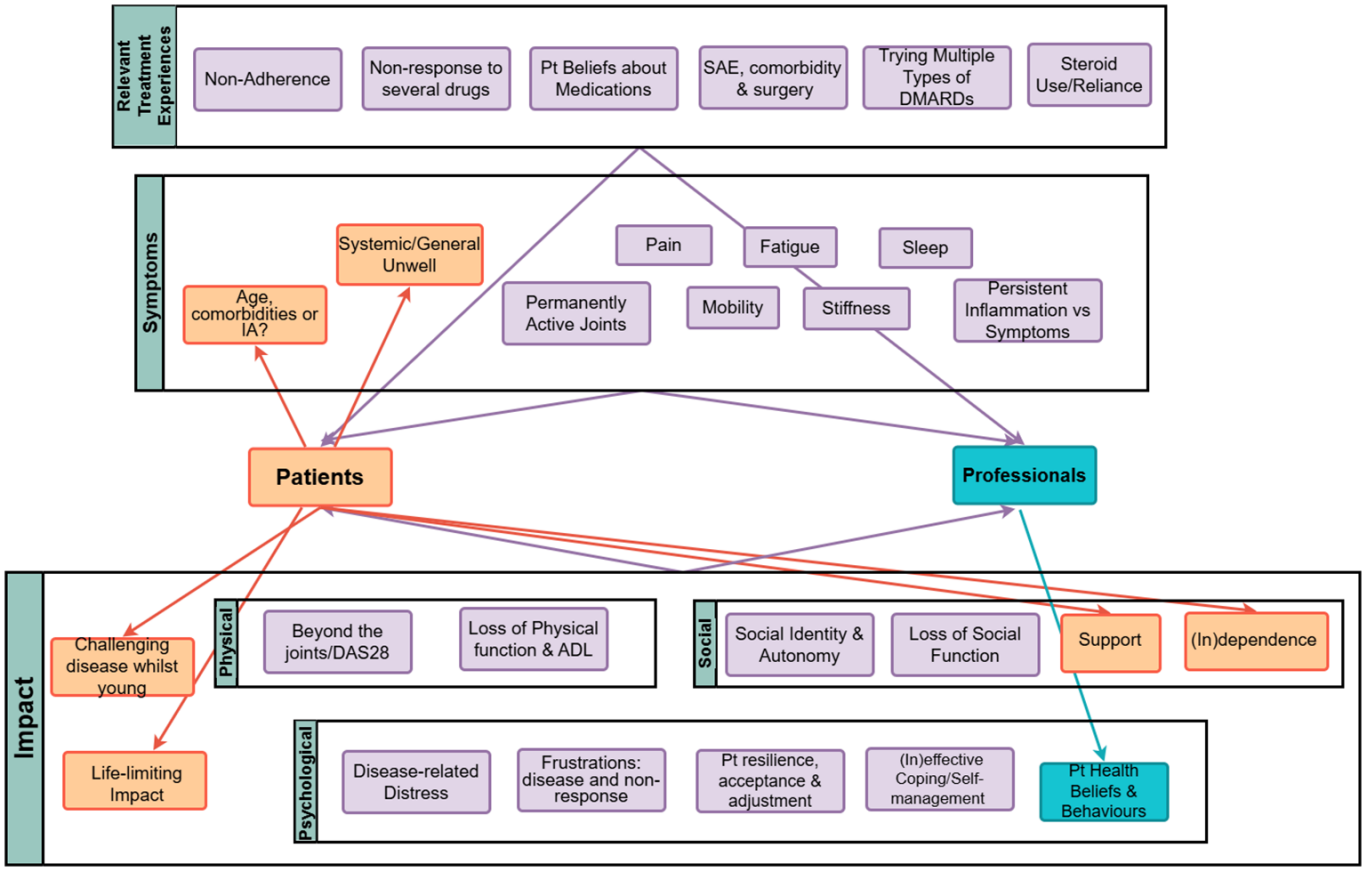
Thematic map of characteristics of RD/PPES identified by patients and health care professionals. Patient‐specific components in orange, HCP‐specific in blue, with shared components in purple grouped into themes. ADL, activities of daily living; DAS28, Disease Activity Score‐29 joints; DMARDs, disease‐modifying anti‐rheumatic drugs; IA, inflammatory arthritis; Pt, patient; SAE, serious adverse event.

Themes characterizing RD/PPES are presented in Figure 1 (see Appendix S4) highlighting similarities and differences between patients and health care professionals. Three key themes were identified from both patients' and professionals' experiences of RD/PPES: (1) relevant treatment experiences, (2) symptoms (with or without inflammation) and (3) impact: physical, psychological and social. Relevant treatment experiences covered elements of treatment that were pertinent to characterizing RD/PPES. Specific common physical and mental symptoms were identified that occur in RD/PPES, with or without Inflammation. Finally, the biopsychosocial impact that these treatments and symptoms had on people living with RD/PPES were detailed. These themes covered 28 subthemes that would be considered as components characterizing RD/PPES, with 6 being patient‐specific and 1 health care professional‐specific. Through the framework analysis, patterns across the data and participants were explored (see Appendix S5).

### Relevant treatment experiences

This theme is comprised of six subthemes (see Table 3). Both patients and health care professionals described the core of RD/PPES as trying multiple disease‐modifying anti‐rheumatic drugs (DMARDs) and still experiencing non‐response (Subtheme 1.1). Non‐adherence to both pharmacological and non‐pharmacological treatments (Subtheme 1.2) was described as a potential reason why people were not responding to medications as the required dosage/frequency was not being followed. Reasons for non‐adherence were linked to both beliefs about medications and patients' broader health beliefs and behaviours, such as coming off medications for surgery or during acute illnesses. Non‐adherence was mentioned more frequently by the paediatric/adolescent health care professionals and considered as an important factor before labelling as refractory to treatment.

**TABLE 3:** Illustrative accounts for Theme 1: Relevant treatment experiences.

Theme	Subthemes	Patients' accounts	HCPs' accounts
(1) Relevant Treatment Experiences	1.1. Trying multiple types of DMARDs	‘I've had so many drugs, so many different trials and things, I mean there was I think a couple on here that, erm, hadn't been used at the [anonymous hospital] when I went on them’ PAT2C	‘double or triple therapy with synthetic DMARDs, present or past…usually the anti‐TNF ones and then going into other classes of biologics and still very active disease’ HCP2A
	1.2. Non‐adherence	‘I think your age makes a massive difference cos you don't actually realise how important it is to take your medication every day and at the same time every day for it to be effective’ PAT1A	HCP7E: ‘So yeah, treatment does range hugely from people you know never miss a dose of their treatment (agreement) to those who probably (HCP3E: never take a dose) (laughter) haven't taken higher than 20%’
	1.3. Drugs not working at all versus losing effectiveness over time	‘You can be on [medication] for quite some time and then suddenly it either make you feel really ill or just stop working altogether, you know, which it sounds, erm, [the non‐response] was hard to take’ PAT2C	‘if the treatment has failed and given you no response, I would say that has primary inefficacy. You've tried [the drug] but it's done no benefit. If you've been put on a drug that's been effective and then worn off, you develop anti‐bodies and it becomes less effective so that's secondary inefficacy’ HCP6D
	1.4. Interference of comorbidities, side effects, surgery and infections	‘Gold injections was like a miracle…but the reaction was really bad. [Enbrel] I tried them for about a few weeks but I used to constantly get a sore throat and colds and all that, I had to stop that…when I had a surgery I had to stop [Infliximab]’ PAT2B	‘you may not be able to give [patients] an anti‐TNF because they've got a history of cancer, or you may not be able to give Tocilizumab because they've got diverticulitis’ HCP6D
	1.5. Steroid use/reliance	‘[steroid] injections is just like a miracle worker, that is, it's so good… Wish they'd come up with one that is just like that, cos it's definitely better than anything else I've ever tried’ PAT3A	‘the fear of steroids running [wearing] off, [patients are] actually quite anxious about even though you're giving them the new, the biologic, [patients are] actually just they're much more concerned about their steroid and just getting their steroid’ HC5B
	1.6. Beliefs about medications: necessity versus concerns	‘It does worry me sometimes, whatever medication you are taking for RA is doing some damage to something somewhere’ PAT1C	‘there are big dilemmas about medication. That does come up in the refractory group in the sense of limited benefits, knowing that they're medications with, with significant side effects, and you know therefore do I continue?’ HCP6B

Not responding to several drugs confirmed perceptions of the medications not working at all (primary inefficacy) versus losing effectiveness over time (secondary inefficacy) (Subtheme 1.3) which also fitted with the unpredictable nature of treatment response and the emotional impact that the medications not working can have, especially when patients had been adherent. When prescribed a drug that worked for them, the interference of comorbidities, adverse side effects, surgery or infections (Subtheme 1.4) were reasons why patients had stopped medications in agreement with clinicians or lost their previously good response. Both these subthemes could reduce the number of available treatment options, and resulted in the likelihood of receiving the label of RD.

Dependency on steroid injections or tablets (Subtheme 1.5) was identified as important to relieve symptoms, due to their efficacy in reducing pain and swelling, with patients finding steroids more effective than DMARDs at reducing symptoms. Beliefs about medications (Subtheme 1.6) were important with patients balancing their fear/anxiety of the side effects with the need to take medications to control the disease.

### Symptoms (with or without inflammation)

This theme contained nine subthemes identified as important symptoms that occur for those with RD/PPES, either with or without inflammation (see Table 4). Symptoms do not occur in isolation and are often interlinked with one affecting another. These can occur in both the presence and absence of active inflammation (Subtheme 2.1) which can be equally debilitating. Both patients and health care professionals described the variety of ways in which joints were involved: permanently swollen, damaged or replaced joint(s), affecting one or two joints (Subtheme 2.2). Accrued damage such as erosion(s), deformity(ies) or restricted movement which may be painful was also discussed when assessing joints or determining treatment control/response. The affected joints could be the feet or ankles which tend to be missed out for the DAS28 assessments and are important for patients, especially PolyJIA.

**TABLE 4:** Illustrative accounts for Theme 2: Symptoms (with or without Inflammation).

Theme	Subthemes	Patients' accounts	HCPs' accounts
(2) Symptoms (with or without Inflammation)	2.1. Persistent inflammation versus symptoms	‘your inflammation levels are low or non‐existent but you still getting problems, pain problems’ PAT5C	‘the refractory people who they're responding erm well from a biological perspective, but they still have a lot of symptomatology and then there's a group of people who are “true biologic failures.” They still have elevated inflammatory markers and they have actively swollen joints and if you scan on ultrasound the joints light up and that's despite drugs’ HCP1B
	2.2. Permanently swollen/damaged/affected joint(s)	‘with the exception of my elbow, err which is err a joint that causes me significant pain err, stiffness and got this swelling and historical damage, I'd say that [PolyJIA] been controlled fairly well’ PAT2D	‘some patients, yes, may never have that inactive disease because they've already had the damage, so you'll never achieve that target’ HCP5D
	2.3. Pain	‘it's the pain cos it's constant and technically, by rights I should be used to it but every time they seem to cure pain in one area…another joint seems to go out somewhere or it changes’ PAT4C	‘[patients] might say: ‘stabbing’ or ‘throbbing’ or they often have, well sometimes will have quite distressing images maybe associated with their pain. Will sometimes describe the pain as sort of something they want, just want to get rid of, an unwelcome guest’ HCP1A
	2.4. Fatigue	‘I've suffered more from fatigue and exhaustion than I've ever suffered before and that's been quite a surprise to me’ PAT5D	‘the fatigue that a lot of patients describe, that sometimes goes away with biological suppression of the active disease, can persist and we're not very good at managing [fatigue] at all’ HCP3B
	2.5. Sleep	‘I'm so stiff I get very little sleep: I find it difficult to turn over in bed and to get out and to go to the toilet or in the morning when I want to get up it's a really painful issue’ PAT5C	‘sleep again is a big issue like they will say: “I can't sleep, I'm tired all the time because…usually in relation to pain’ HCP5D
	2.6. Mobility	‘I can't walk downhill, I can't walk downstairs, can't walk upstairs, erm, yeah, can't really walk very much’ PAT3B ‘getting dressed, and I take ages to get in the shower and, erm, like personal things. [RA has] really has slowed me up over the last few years so obviously it's affected my quality of life a great deal’ PAT4D	‘shoulders, often something that doesn't come up, but they'll often complain about in a peripheral, like hand, wrist symptoms and they'll have problems there, but when you ask [patients] to erm reach over their head or do any sort of functional activities above sort of shoulder‐level, and they can't do that and sometimes they're not even aware of that’ HCP5C
	2.7. Stiffness	‘[my RA is] definitely active, there's no doubt about that, erm, it causes, erm, principally stiffness either in the mornings or from any prolonged period of sort of sitting down or standing up, for that matter’ PAT6D	‘early morning stiffness can last all day’ HCP8C ‘“Oh, my feet are a little bit stiff, I can't get the same movement in some of the joints” etc. or “My toes are stiff”’ HCP1C

Theme	Subthemes	Patients' accounts	HCPs' accounts
(2) Symptoms (with or without Inflammation)	2.8. Systemic/general unwell	‘I was really quite tired and felt a bit fluey, which, you know, which I often feel like with rheumatoid. I felt like I was constantly getting over flu, I think is the best way to describe it’ PAT3B	
	2.9. Age, comorbidities or IA?	‘[RA's] stopped me doing things that I want, used to do, but erm I just put that down to my age as well’ PAT8D ‘my eyes have been straining a lot but is that because of my arthritis, is it because of my medication, or is it just because I've always, I've worn glasses since I was 11?’ PAT1A	

Another prominent symptom is Pain (Subtheme 2.3), with many participants mentioning it needed to be addressed in their daily lives. Patients reported pain occurring every day regardless of activity with a knock‐on effect on fatigue and mood and is the reason for physical or social activity restrictions. Fatigue was commonly described due to ‘the body fighting the condition’, living with pain or as a side effect from medication (Subtheme 2.4). For some patients this discomfort was unexpected and sometimes a separate entity that is difficult to manage or receive support for. Health care professionals shared this sentiment. Patients consistently mentioned that their fatigue seems to be linked to pain, and this included cognitive fatigue affecting concentration and memory. Sleep difficulties were a separate complaint (Subtheme 2.5), due to the pain and stiffness in their joints causing the inability to sleep comfortably or due to lack of physical activity.

The combination of pain, stiffness and fatigue may result in reduced mobility in some patients, for example, walking long distances, navigating stairs or difficulties using public transport (Subtheme 2.6). Consequently, these mobility problems were linked with other categories such as impaired physical and social functioning with people reporting their IA slows them down. Patients indicated that persistent stiffness (Subtheme 2.7) was detrimental and lasted longer than 30 min in the morning that is routinely asked by clinicians. Two patient‐specific categories were identified. Firstly, patients reported they had a general feeling of unwellness similar to flu that they attributed to their Inflammatory Arthritis (Subtheme 2.8). Secondly, difficulty ascribing their symptoms to age, comorbidities or their rheumatic condition (Subtheme 2.9), further highlighting the confounding influence of multimorbidity in RD/PPES.

### Physical, psychological and social impact

There were physical, psychological and social ramifications and influences from experiencing RD/PPES that were highlighted in the next three categories which covered 11 subthemes (see Tables 5, 6, 7), with an over‐arching patient‐only subtheme that represented the complete overall life‐limiting impact across these subthemes and emphasized the wide‐ranging influence of RA/PolyJIA in all aspects of life (Subtheme 3.1). This was also seen in questionnaire scores which indicated a moderate quality of life. The psychological impact could either perpetuate ongoing symptoms and contribute negatively to patients' quality of life or be protective and allow people to experience good quality of life despite RD/PPES. People with PolyJIA reported greater variety in their experiences compared to people with RA, in particular the challenges of living with a long‐term illness at an early stage in their lives (Subtheme 3.2).

**TABLE 5:** Illustrative accounts for Theme 3: Impact and (a) Physical.

Theme	Subthemes	Patients' accounts	HCPs' accounts
(3) Impact	3.1. Life limiting	‘when [daughter] was younger, can't sit on the floor, you can't do certain things that they want you to do, which can be quite frustrating…constantly having to cancel appointments cos you don't feel well enough to be able to get out’ PAT6A ‘I had to change, I used to be an auxiliary nurse, years ago and I had to give that up’ PAT5A	
	3.2. Challenging disease whilst young	‘saying to people like, “Arthritis”, people are like, “What? You're not 80” or like those connotations and not wanting to feel like I'm 80 as well, like you know, like those days when I'm swollen and I'm sore and I think ugh, I shouldn't feel like this at this age’ PATT11D	
(3a) Physical Impact	3a.1. RA/JIA impacts beyond the joints/DAS	‘I lost weight cos [PolyJIA] affected not just my knees and my legs, but also affected my mouth and one of my arms’ PAT9D ‘Sjögren's syndrome, erm, Barrett's oesophagitis, so, those are all a result of, allodynia, fibromyalgia, those are all a result of RA, which have affected me. My eyes now are quite affected, I can only look at a screen for so long, or a book or reading, my eyes get stressed’ PAT4B	‘we run the risk as rheumatologists of being too joint focused and not considering the other impacts, erm, for example, low mood, poor sleep’ HCP3C
	3a.2. Loss of physical function and activities of daily living	‘daily activities are hard, you know, cos you do everything with your hands (laughs) it's ridiculous really…if I could get given a new set of hands, I would be alright’ PAT4A ‘[RA's] stopped me from doing a lot of stuff that I used to do. I have to think twice about certain [activities], places to go’ PAT7D	‘problems that are to do with living so how those things then impact on work and emotion and social life and all those sorts of things’ HCP2C

**TABLE 6:** Illustrative accounts for Theme 3: (b) Psychological.

Theme	Subthemes	Patients' accounts	HCPs' accounts
(3b) Psychological Impact	3b.1. Frustrations: disease and non‐response	‘how can [PolyJIA] be so difficult to treat, it's inflammation! It makes you think well, how can you not treat my disease?’ PAT6C	‘often outcomes aren't as good as we'd like and, erm, it's frustrating for the patient and frustrating for us at times that there's not more that we can do for these patients’ HCP2B
	3b.2. Patient attitude, resilience, acceptance and adjustment	‘[RA] is controlled compared to how I used to be cos I used to be in a bad, bad way. That's why I say I'm happy, it's just the fact that I can move around’ PAT10D ‘my life would be different to how it is now having had the disease, without a doubt. But that's not necessarily a terrible thing’ PAT1B	‘[patients] have been told that their bloods and everything are looking good, but they're absolutely feeling awful in themselves and struggling to cope with [symptoms]. So, that makes it harder for them to actually accept and manage and move forward with condition’ HCP7C ‘there's a lot of denial…there's equally those that don't complain, those silent ones that put two fingers up to their illness and don't engage with you and try and avoid it’ HCP6E
	3b.3. Effective or ineffective coping/self‐management	‘I know certain positions to put myself in to feel better; I know ways to pace myself; I know what to do, and then this new joint that I don't know how to manage…I haven't worked out that pacing yet (hmm) to this new joint, erm, and so yeah, that's kind of disheartening’ PAT11D	‘sometimes people aren't doing a paced activity or exercise programme, mainly because they're perhaps frightened of how much they are able to do or they're worried about causing a flare‐up, so that's despite having advice from physios erm regarding, how to actually manage exercise appropriately…sometimes people have got very fixed ideas on diet’ HCP7C
	3b.4. Disease‐related distress and negative psychosocial impact	‘I haven't even gone for a blue badge to be quite honest with you. It's stressful for me going to my doctor and asking him to fill out a form, is just too much’ PAT2A ‘affects you mentally a lot you can get depressed…What am I going to do in the future?’ PAT2B	‘late effects of having arthritis when you're three, goes into remission by eight, flares at age 14 with major psychological, you know, but might not seem like dramatic inflammation but huge psychological, you know, it's almost like a late effect of, like talking cancer late effects’ HCP3E
	3b.5. Patient health beliefs and behaviours		‘parents have got their own histories of difficult medical, kind of ailments, that's going to very much affect how a young person sees their wellness score or how they feel’ HCP3D

**TABLE 7:** Illustrative accounts for Theme 3: (c) Social.

Theme	Subthemes	Patients' accounts	HCPs' accounts
(3c) Social Impact	3c.1. Affected social identity and autonomy	‘I just think people will just get fed up with you saying: “Oh, this hurts and that hurts”. So, in the end you say nothing because if you don't want people to think: “Oh god, I'm not going out with her today, she's nothing but a moaner”’ PAT3D	‘patients that are isolated can be high risk if they don't have good social support networks and I have a particular concern about isolated men and suicide risk’ HCP2C ‘being different from their peers, not being able to do what their peers do. Struggling with school, exams’ HCP4D
	3c.2. Loss of social function	‘Eating out is very difficult but I would rather do that than lose my friends’ PAT4B	‘having friends, that sort of thing. So, in particular, not being able, not taking part in PE or sports or playing out with their friends’ HCP4E
	3c.3. Independence versus dependence on others	‘you can't wash yourself, you can't wipe yourself sometimes when you go to the toilet cos your hands are like clawed up or sore…to me it's the indignity of not being able to look after yourself’ PAT1C	
	3c.4. Support from partners or parents	‘I live on me own, erm, if I was, say, living with me parents or I was married, it might be a different thing cos there'd be somebody there, I could discuss it’ PAT4C	

The physical impact of RD/PPES are split into two subthemes. The first subtheme included elements such as related comorbidities, involvement of other joints such as feet or jaw (especially for those with PolyJIA) and other symptoms, for example, problems eating, highlighting the involvement of joints and areas not captured by disease activity measures such as the DAS28‐joint count, for example, feet/ankles and eyes (Subtheme 3a.1). A recurring element for health care professionals was how restrictive the DAS28 score is in assessing RD/PPES in RA/PolyJIA, and that wider factors need to be considered such as persistent foot inflammation, erosions and physical activity ability. The second category pointed to the inability to perform desired activities/tasks, for example, cooking, work and restrictions in performing activities of daily living, for example, washing/dressing, cooking and poor physical function usually in hand strength/dexterity and reflecting the impaired quality of life (Subtheme 3a.2). People described how they could not do things that they used to, with daily activities and social events needing to be planned to account for their physical restrictions.

The psychological impact represented five subthemes. Frustration was the most common emotion that both patients and health care professionals expressed regarding RD/PPES (Subtheme 3b.1). Frustrations could be about activity limitations, drug routine and not being able to live a normal life. Additionally, feeling fed up as not responding to treatment or finding the right medication, which health care professionals found equally frustrating. In contrast, some patients had a resilient attitude and just ‘get on with it’. Many had accepted the illness and life limitations (Subtheme 3b.2). Patients would also describe that their disease was not fully controlled by medication but was manageable as they were doing better than in the past or compared to others. Health care professionals also reported that patients would say their condition was acceptable despite ongoing symptoms which rheumatologists wanted to further treat, therefore describing their patients as ‘stoic’ to reflect this resilience. However, some discordant views were expressed where health care professionals perceived that patients had not accepted the illness or were in denial about their condition and limitations.

These attitudes and beliefs may affect whether patients undertook (in) effective coping or self‐management activities (Subtheme 3b.3). For example, some used active coping strategies such as pacing and planning activities or exercises to keep joints mobile and taking pain medication when required. Passive coping involved avoiding activities or situations and not maintaining strength/dexterity as worried exercises could worsen symptoms. Although this type of coping was justified by patients stating they only avoided activities that would aggravate their condition/joints rather than being in denial or avoiding taking medication. Key to self‐management and coping was patients knowing their limitations and what they can do. Some would acknowledge that they were ‘over‐doing things’ through undertaking activities beyond their limits but would rather suffer the consequences than not complete the physical or social activity.

Alternatively, if a new joint was affected or symptoms were worsening, this could be disheartening and cause distress. Negative psychosocial impacts included low mood, loss of interest in activities, worry about future deterioration and emotional distress, including grieving the life they had before the Inflammatory Arthritis onset (Subtheme 3b.4). Depression or anxiety could be separate comorbidities but often this was more related to elements of having RA/PolyJIA, such as experiencing ongoing/worsening or relapsing–remitting disease or fears of needing surgery or being in constant pain. Disease‐related distress and burden were also mentioned such as the stress of having to coordinate care between GPs and their rheumatology teams, attending multiple/frequent appointments or proving eligibility for government assistance that they are entitled to.

Experiences of the psychosocial impact due to that ongoing inflammation and symptoms was reported by several health care professionals who mentioned patients described negative feelings and low mood when explaining symptoms or problems to their rheumatology team and being defined by inflammatory arthritis with feelings of despair and being fed up. Patient health beliefs and behaviours was the only health care professional‐specific category (Subtheme 3b.5) and included perceptions of patients understanding of their condition, health literacy and the illness representations that they hold that affected their health behaviours. Paediatric health care professionals mentioned the role that parent's beliefs can play in young people's health understanding and subsequent behaviour and the resulting impact these beliefs and behaviours may have on their disease progression.

Finally, the social impact of RD/PPES covered four subthemes. Social identity can be affected as people cannot engage in activities, they used to be able to do, and their Inflammatory Arthritis causes them to be isolated and defined by their illness (Subtheme 3c.1). This isolation and loneliness due to lack of a good support network was identified as a risk for psychosocial difficulties. Furthermore, a lack of autonomy as feeling dependent on others or the lack of opportunities to develop socially for those with PolyJIA may cause them to withdraw. Impaired social participation/function was a problem (Subtheme 3c.2), with patients stating they had difficulties making and keeping friends and to socialize in different ways. Paediatric health care professionals reported reduced social functioning more frequently than adult professionals due to the influence and importance of friendships in children and adolescents. Some patients experienced no problems socially because of their RA/PolyJIA, if they had a good group of friends who understood their condition and restrictions.

Reduced independence and increased dependence on others (Subtheme 3c.3) in terms of practical help and emotional support was reported by patients. This assistance is part of the day‐to‐day reality of managing RA/PolyJIA and perhaps not routinely discussed in clinics. There was a struggle between wanting to remain independent whilst also needing to be dependent on others for certain activities that they could no longer do. This struggle could cause distress that they could no longer look after themselves. In contrast, some were thankful to have a partner, who provided ongoing support both physically and mentally. The type of support provided by partners or parents (Subtheme 3c.4) ranged from giving comfort, practical help with household chores or shopping or administering medications or help to attend social events, thereby protecting quality of life. The role of a supportive partner was most evident in two unmarried men interviewed as they experienced the negative impacts on functioning and quality of life, possibly due to not having a helpful partner and they felt isolated.

## DISCUSSION

The aim of this qualitative study was to explore patients' and health care professionals' understanding and experiences of RD/PPES in RA/PolyJIA from both of their perspectives. Three key themes were identified from both patients' and professionals' experiences of RD/PPES: (1) relevant treatment experiences, (2) symptoms (with or without inflammation) and (3) impact: physical, psychological and social. These themes included 28 specific subthemes that would be considered as components characterizing refractory disease, of which most subthemes were common to both patients and health care professionals, whilst six were patient‐specific and one was health care professional‐specific.

The areas identified here are important characteristics of RD/PPES and fit with priorities identified in a qualitative study with patients diagnosed with RA for clinical outcomes after pharmacological interventions (Sanderson et al., 2016). Sanderson and colleagues highlighted that the severity, effect and coping of these priorities needed to be considered as was found in this current study in this specific subgroup of RA/PolyJIA through the symptoms and impact themes. Living with RD/PPES causes specific disease‐related distress, distinct from general depression and anxiety, as highlighted throughout the patients' accounts, covering physical‐, emotional‐, social‐, treatment‐ and health care‐related distress (Silke et al., 2021). An important element of psychological impact was the influence of patients' health beliefs and understanding of their disease (Horne et al., 2019; Leventhal et al., 2016), which had wide‐reaching effects on self‐management and treatment adherence. Health care professionals need to explore these beliefs to dispel misconceptions, align treatment expectations and readjust disease management. However, this can be difficult to implement during consultation due to discordances in expectations and beliefs between patients and clinicians (Berenbaum et al., 2014).

A clear protective factor was the role of a supportive partner. Those without significant others seemed to have more problems in daily life, experiencing loneliness and inability to do housework, including reduced social functioning. Both patients and HCPs reported ‘getting on with it’ and being resilient which could be perceived as positive. There may be problems when showing resilience or stoicism, for example, the ones that do not complain, when in fact may require further support or treatment to prevent worsening/suboptimal outcomes (Gwinnutt et al., 2019). This group may require increased vigilance by multi‐disciplinary rheumatology teams to optimize clinical outcomes.

### Strengths and limitations

To our knowledge, this is the first and currently only qualitative study to explore RD/PPS in IA across the life course transdiagnostically in RA/PolyJIA, including both patients and health care professionals, which has enabled an in‐depth analysis into this important but poorly understood subset of patients that are missing from other refractory/difficult‐to‐treat research initiatives (Buch et al., 2021; Nagy et al., 2020). This study has provided novel insights into the characteristics of RD/PPES in RA/PolyJIA to contribute to a comprehensive biopsychosocial understanding for both clinicians and researchers, whilst identifying areas important for patients. Elements relevant to those with PolyJIA, paediatric health care professionals and males were highlighted throughout and in the accounts presented, in particular regarding the psychosocial impact. A clear strength is the large sample size of both patients and multi‐disciplinary health care professionals, who were interviewed to obtain multiple perspectives about RD/PPES, across RA/PolyJIA and adult/paediatric care. The transdiagnostic approach taken here may increase the transferability of the findings which could be applied to RD/PPES in other inflammatory arthritis conditions such as psoriatic arthritis or spondyloarthropathies in both paediatric and adult populations.

The validity and credibility of findings were established during analysis and reporting by following the JARS guidelines, including a thematic map (O'Brien et al., 2014). Also, transparency of the analysis was achieved by using direct data from transcripts from the framework and reporting, providing specific evidence of how the data led to the interpretation and theme allocation (Gale et al., 2013; Leal et al., 2015). This study also included additional views of health care professionals such as a pharmacist, podiatrist and social worker, whose perspectives are often missing (Dures et al., 2014; van Tuyl et al., 2008), therefore taking a wider multidisciplinary team approach aligning with the Rheumatology workforce (British Society of Rheumatology, 2021). Furthermore, other less researched perspectives of adults with PolyJIA and those with PPES, despite controlled inflammation, were incorporated through a targeted recruitment strategy employed in this study.

There are some limitations to be acknowledged. Patients and health care professionals from England only (predominantly London) participated in this study and may therefore not be representative of experiences across the United Kingdom. Identifying patients based on clinical data was problematical given the levels of missing data, in particular the DAS28 scores, important for inclusion. Missing DAS28 scores have been found in a national audit due to clinicians emphasizing they collect this data more for patients with ‘typical’ or severe symptoms/disease of Inflammatory Arthritis due to the requirement for treatment escalation (Yates et al., 2020). This reason may explain why recruiting those with PPES was more difficult to screen and recruit as DAS28 scores were not conducted in patients with less severe presentation and are less frequently conducted in adult PolyJIA patients.

There are advantages and disadvantages to face‐to‐face versus telephone methods (Saarijärvi & Bratt, 2021), and the depth of data obtained may have been compromised as non‐verbal communication by telephone interviews could not be included. Likewise, the depth of data in focus groups can be limited as some participants may dominate (Acocella, 2012) the information sharing. However, by combining multiple data collection methods each reveals different, complementary elements to contribute a comprehensive understanding and address shortcomings of a singular methodology aligning with a pragmatic epistemological approach (Johnson & Onwuegbuzie, 2004; Lambert & Loiselle, 2008).

Health psychologists can help support rheumatology multi‐disciplinary teams to tackle some of the psycho‐social symptoms and impact patients' experience as reported during the interviews through delivering or developing complementary non‐pharmacological interventions and/or training, for example, to rheumatology nurses in line with patient preferences to deliver psychological support and/or counselling (Dures et al., 2016). For example, patient education self‐management interventions, to be embedded in routine clinics, or provide communication training to incorporate these topics in clinical consultations, in close collaboration with patients to ensure the interventions meet their needs (McBain et al., 2018).

## CONCLUSION

RD/PPES in RA/PolyJIA can be summarized as persistent symptoms with biopsychosocial impact, either in the presence or absence of inflammation despite treatment with multiple DMARDs. As found through this analysis, the specific biopsychosocial symptoms and impacts of RD/PPES pertain to pain, fatigue, stiffness, joint involvement and the physical, psychological and social impact of persistent symptoms including disease‐related distress, mobility and independence. Also, wider influential factors such as comorbidities, non‐adherence, health/medication beliefs and behaviours and social support are part of RD/PPES. This analysis has highlighted specific symptoms and impact that could be targeted through psychosocial interventions by multi‐disciplinary health care professionals (Chaplin et al., 2024), in particular health psychologists or clinical psychologists specializing in musculoskeletal health.

## AUTHOR CONTRIBUTIONS

**Hema Chaplin:** Conceptualization; methodology; formal analysis; visualization; writing – original draft; writing – review and editing; project administration; funding acquisition; investigation. **Carol Simpson:** Writing – review and editing; supervision. **Kate Wilkins:** Supervision; writing – review and editing. **Jessica Meehan:** Formal analysis; writing – review and editing. **Nora Ng:** Investigation; resources; writing – review and editing. **James Galloway:** Investigation; resources; writing – review and editing. **Ian C. Scott:** Writing – review and editing; resources; investigation. **Debajit Sen:** Investigation; resources; writing – review and editing. **Rachel Tattersall:** Investigation; resources; writing – review and editing. **Rona Moss‐Morris:** Writing – review and editing; supervision. **Heidi Lempp:** Conceptualization; methodology; formal analysis; writing – original draft; writing – review and editing; visualization; supervision; funding acquisition. **Sam Norton:** Conceptualization; methodology; formal analysis; writing – original draft; writing – review and editing; visualization; supervision; funding acquisition.

## FUNDING INFORMATION

This paper represents independent research funded by the National Institute for Health Research (NIHR) Maudsley Biomedical Research Centre at South London and Maudsley NHS Foundation Trust and King's College London, in the form of a PhD Studentship (IS‐BRC‐1215‐20018) for the first author (HC). The views expressed are those of the author(s) and not necessarily those of the NHS, the NIHR or the Department of Health and Social Care. SN was partially supported by funding from MQ and Versus Arthritis (MQF16IP\100018). HL is currently supported by the UK Medical Research Council (UKRI) for the Indigo Partnership (MR/R023697/1) and Adolescents' Resilience and Treatment nEeds for Mental health in Indian Slums (ARTEMIS): (MR/S023224/1) awards. ICS is funded by the NIHR (Advanced Research Fellowship—NIHR300826).

## CONFLICT OF INTEREST STATEMENT

None.

## ETHICS STATEMENT

Full NHS ethical approval granted by London—Hampstead Research Ethics Committee (18/LO/1171, June 2018).

## Supporting information

Appendices S1–S5.

## Data Availability

Majority of data is contained within the Supplementary File (Framework) and further data is available upon request from the corresponding author (HC).
