# Supplementary material for: Characteristics of refractory disease and persistent symptoms in inflammatory arthritis: Qualitative framework analysis of interviews with patients and health care professionals
Source: Br J Health Psychol. 2025 Jan 8;30(1):e12780. doi: 10.1111/bjhp.12780 (PMC11707814; doi:10.1111/bjhp.12780)
Supplement: Supplementary file 1 — Appendices S1–S5. [file BJHP-30-0-s001.zip › S1_-_Additional_Methodological_Information_V3_19.12.2024.docx]

**S1 - Additional Methodological Information**

**Researcher Description**

Based on HC’s previous patient involvement work in Adolescent and Young Adult Rheumatology, she identified that there was a gap and need for health psychology research projects integrating physical and mental health in Rheumatology. Through HC’s PhD Studentship (which this current study forms part of), she took a transdiagnostic approach to include JIA as well as RA as Refractory Disease also affects this younger population from her previous experience working in Adolescent Rheumatology. HC felt it was important to explore Refractory Disease through a wider approach with a Health Psychology lens instead of conducting primary research from a purely inflammation driven perspective which has influenced the current research.

**Epistemological Stance and Data Analysis**

A pragmatic epistemology approach allows the researcher to conduct analysis underpinned with an orientation to understand the importance of participants’ perspectives in the context of their lives and the resulting empirical and practical consequences, whilst representing participants accounts accurately (Johnson & Onwuegbuzie, 2004). This approach is necessary to address the study aim.

Qualitative research can systematically generate insights about people’s experiences, beliefs, and attitudes, not always expressed during clinical appointments, which can explore and address complex and challenging areas such as RD/PPES (Kelly et al., 2021). A quantitative questionnaire, with open-ended questions, could have been considered as a method of data collection to explore characteristics of RD/PPES, but this is not appropriate given the exploratory nature of this study and gap identified in the understanding of RD at the time (Young, 2015) and lack of literature or appropriate theory to base such a questionnaire on.

Framework analysis was used because it is grounded in the original participant accounts, is a dynamic, systematic analytic process, and enables comparisons between, and associations within, cases to be made (Ritchie & Spencer, 1994; Srivastava & Thomson, 2009). This analytic approach can also be used on data collected from individual interviews and focus groups (Goldsmith, 2021) to develop a thematic framework to capture and describe data that identifies similarities and differences.

**Data Collection**

In this study we decided to conduct individual’s interviews with patients and focus groups with HCPs. Interviews and focus groups also may be combined for the purposes of data completeness, as it is assumed that each method reveals different, complementary parts to contribute towards a comprehensive understanding (Lambert & Loiselle, 2008) of the phenomenon studied. For example, individual interviews may be conducted to explore personal experiences (patient perspective), whereas focus groups may be carried out to examine opinions and beliefs about the phenomenon (HCP perspective). There are three broad rationales for combining interviews and focus groups (Lambert & Loiselle, 2008) that have been implemented in this study: 1) pragmatic reasons such as participants’ preference, 2) the need to compare and contrast participants’ perspectives as explored here through framework analysis, and 3) due to practical reasons such as limited resources (e.g. time and money) during this PhD Studentship and participant preference/availability as detailed below.

Focus groups were deemed an appropriate practical solution for engaging with busy HCPs by capitalising on established group dynamics to stimulate discussion through conducting these at the same time as routine clinical multi-disciplinary meetings (Guest et al., 2017). In reality, these meetings occurred at busy times with no suitable free time alongside the meeting, therefore separately arranged focus groups or one-to-one interviews were conducted depending on HCPs availability and preference. Depending on HCP availability and/or preference, focus groups (aligned with scheduled clinical meetings where possible) or one-to-one interviews (See Table 2) were arranged.

### Table 1: Focus Group Composition

| Focus Group 1 | Focus Group 2 | Focus Group 3 | Focus Group 4 | Focus Group 5 |
| --- | --- | --- | --- | --- |
| Consultant  Consultant  Registrar  Registrar  Pharmacist | Consultant  Specialist Nurse  Consultant  Consultant | Consultant  Consultant  Consultant  Consultant  Consultant  Consultant | Specialist Nurse  Social Worker  Consultant | Registrar  Registrar |

### Table 2: Interview Modality Composition

| Telephone | Face-to-face |
| --- | --- |
| Consultant  Consultant  Consultant  Physiotherapist  Specialist Nurse  Occupational Therapist  Occupational Therapist  Podiatrist | Physiotherapist  Specialist Nurse  Psychologist  Psychologist |

For data collection from patient participants it was anticipated that there may be pressure to provide socially desirable responses and agree with dominant voices in this group setting (Acocella, 2012), and they may not disclose personal or sensitive information (Guest et al., 2017). Therefore, individual interviews would generate a depth of data about their personal experiences of RD/PPES. Depending on patient preference, interviews were conducted face-to-face or over the telephone. Interviews took place between 8^th^ October 2018 and 15^th^ April 2019, lasting between 32-95 minutes, with nine conducted in person and 16 over the telephone. Face-to-face interviews were conducted in private rooms in university buildings (n=8) or the patient’s hospital (D, n=1) depending on patient preference, and telephone interviews were conducted in a private university room in London with the researcher. Participants were not incentivised or compensated to participate aside from travel expense reimbursements.

One additional partner was present during one interview (PAT5A), with the agreement of the patient, who contributed to the interview and therefore provided written informed consent. Although not in the original ethics application, following correspondence with the ethics committee, staff confirmed the inclusion of the partner was acceptable given the person was fully informed (as they provided informed written consent) and their inclusion was not likely to have a negative impact on the study’s results. The inclusion of one partner giving their perspective on the experience of helping their partner with RA manage their RD/PPES could have added value to the findings (Polak & Green, 2016). However, the influence of a partner in the interview can be problematic, due to ethical and methodological challenges (Zarhin, 2018). The contribution of one partner in this study has been unexpected and its value cannot be overstated.

**Data Analysis and Credibility**

Three coders were involved in this analysis, although the PhD researcher (HC) led the analysis primarily with the support of two coders (HL and JM). HL was the second coder and qualitative supervisor providing advice and support throughout this study phase, especially during the conduct and coding of the first two interviews, with more senior qualitative expertise and training. JM focused on primarily coding the HCP focus group transcripts, as part of her MSc dissertation, which HC seconded coded.

Where appropriate, themes and findings were linked back to theoretical concepts (Carroll et al., 2022; Horne et al., 2019; Leventhal et al., 2016; Moss-Morris, 2013) to align identified concepts/characteristics to established theories and utilise accepted terminology to confirm or strengthen study findings and interpretation (Leal et al., 2015).

**References**

Acocella, I. (2012). The focus groups in social research: advantages and disadvantages. *Quality & Quantity*, *46*(4), 1125-1136. <https://doi.org/10.1007/s11135-011-9600-4>

Carroll, S., Moon, Z., Hudson, J., Hulme, K., & Moss-Morris, R. (2022). An Evidence-Based Theory of Psychological Adjustment to Long-Term Physical Health Conditions: Applications in Clinical Practice. *Psychosomatic Medicine*, *84*(5), 547-559. <https://doi.org/10.1097/psy.0000000000001076>

Goldsmith, L. J. (2021). Using Framework Analysis in Applied Qualitative Research. *Qualitative Report*, *26*(6).

Guest, G., Namey, E., Taylor, J., Eley, N., & McKenna, K. (2017). Comparing focus groups and individual interviews: findings from a randomized study. *International Journal of Social Research Methodology*, *20*(6), 693-708. <https://doi.org/10.1080/13645579.2017.1281601>

Horne, R., Cooper, V., Wileman, V., & Chan, A. (2019). Supporting Adherence to Medicines for Long-Term Conditions: A Perceptions and Practicalities Approach Based on an Extended Common-Sense Model. *European Psychologist*, *24*(1), 82-96. <https://doi.org/10.1027/1016-9040/a000353>

Johnson, R. B., & Onwuegbuzie, A. J. (2004). Mixed Methods Research: A Research Paradigm Whose Time Has Come. *Educational Researcher*, *33*(7), 14-26. <https://doi.org/10.3102/0013189x033007014>

Kelly, A., Tymms, K., Fallon, K., Sumpton, D., Tugwell, P., Tunnicliffe, D., & Tong, A. (2021). Qualitative Research in Rheumatology: An Overview of Methods and Contributions to Practice and Policy. *The Journal of Rheumatology*, *48*(1), 6-15. <https://doi.org/10.3899/jrheum.191368>

Lambert, S. D., & Loiselle, C. G. (2008). Combining individual interviews and focus groups to enhance data richness. *J Adv Nurs*, *62*(2), 228-237. <https://doi.org/10.1111/j.1365-2648.2007.04559.x>

Leal, I., Engebretson, J., Cohen, L., Rodriguez, A., Wangyal, T., Lopez, G., & Chaoul, A. (2015). Experiences of paradox: a qualitative analysis of living with cancer using a framework approach. *Psycho-Oncology*, *24*(2), 138-146. <https://doi.org/https://doi.org/10.1002/pon.3578>

Leventhal, H., Phillips, L. A., & Burns, E. (2016). The Common-Sense Model of Self-Regulation (CSM): a dynamic framework for understanding illness self-management [journal article]. *Journal of Behavioral Medicine*, *39*(6), 935-946. <https://doi.org/10.1007/s10865-016-9782-2>

Moss-Morris, R. (2013). Adjusting to chronic illness: time for a unified theory. *Br J Health Psychol*, *18*(4), 681-686. <https://doi.org/10.1111/bjhp.12072>

Polak, L., & Green, J. (2016). Using Joint Interviews to Add Analytic Value. *Qualitative Health Research*, *26*(12), 1638-1648. <https://doi.org/10.1177/1049732315580103>

Ritchie, J., & Spencer, L. (1994). Qualitative data analysis for applied policy research. In A. Bryman & R. G. Burgess (Eds.), *Analyzing Qualitative Data*. Routledge. <https://books.google.co.uk/books?hl=en&lr=&id=46jfwR6y5joC&oi=fnd&pg=PA305&dq=Qualitative+data+analysis+for+applied+policy+research&ots=snEPHKquQV&sig=poSF7aYx0uWLVGZ47OiYzJODseM>

Srivastava, A., & Thomson, S. B. (2009). Framework analysis: a qualitative methodology for applied policy research. *Journal of Administration & Governance*, *4*(2), 72-79.

Young, A. (2015). *Minutes from ARUK clinical study group refractory disease workshop: Prevalence of refractory disease in the UK*. ARUK clinical study group refractory disease workshop.

Zarhin, D. (2018). Conducting Joint Interviews With Couples: Ethical and Methodological Challenges. *Qualitative Health Research*, *28*(5), 844-854. <https://doi.org/10.1177/1049732317749196>
