# Supplementary material for: Characteristics of refractory disease and persistent symptoms in inflammatory arthritis: Qualitative framework analysis of interviews with patients and health care professionals
Source: Br J Health Psychol. 2025 Jan 8;30(1):e12780. doi: 10.1111/bjhp.12780 (PMC11707814; doi:10.1111/bjhp.12780)

### Supplementary Information S2: A) Patient Interview Schedule


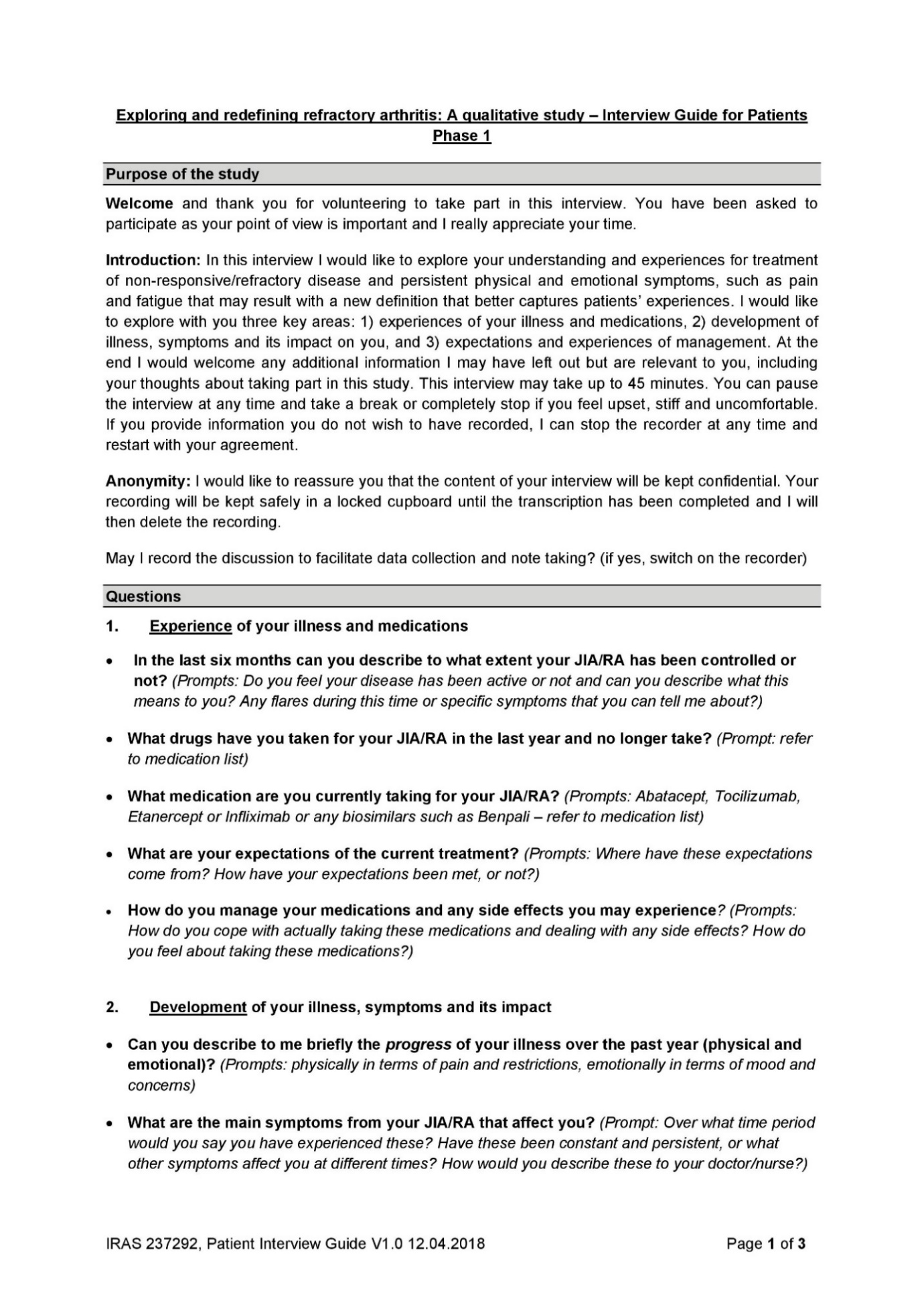


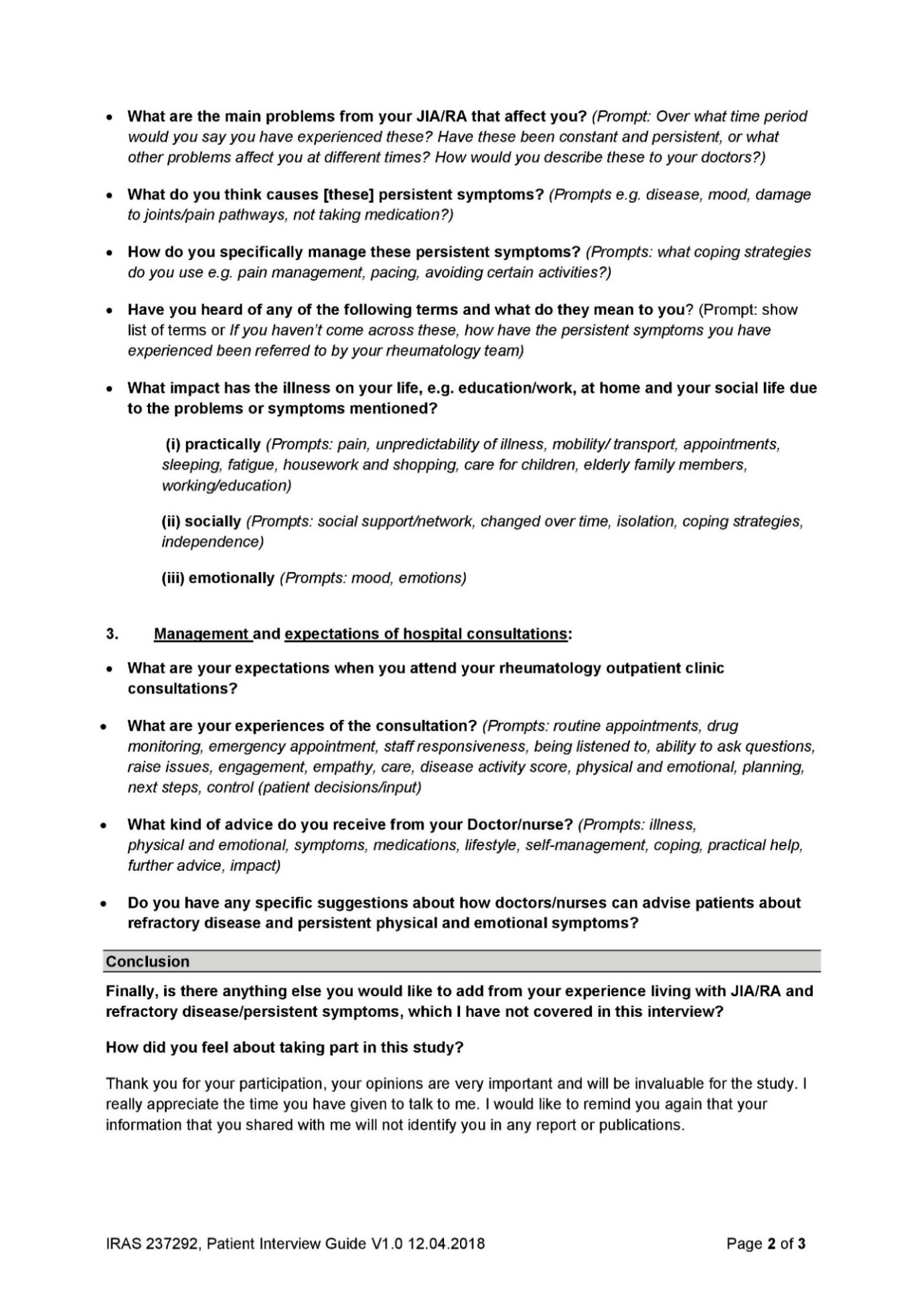


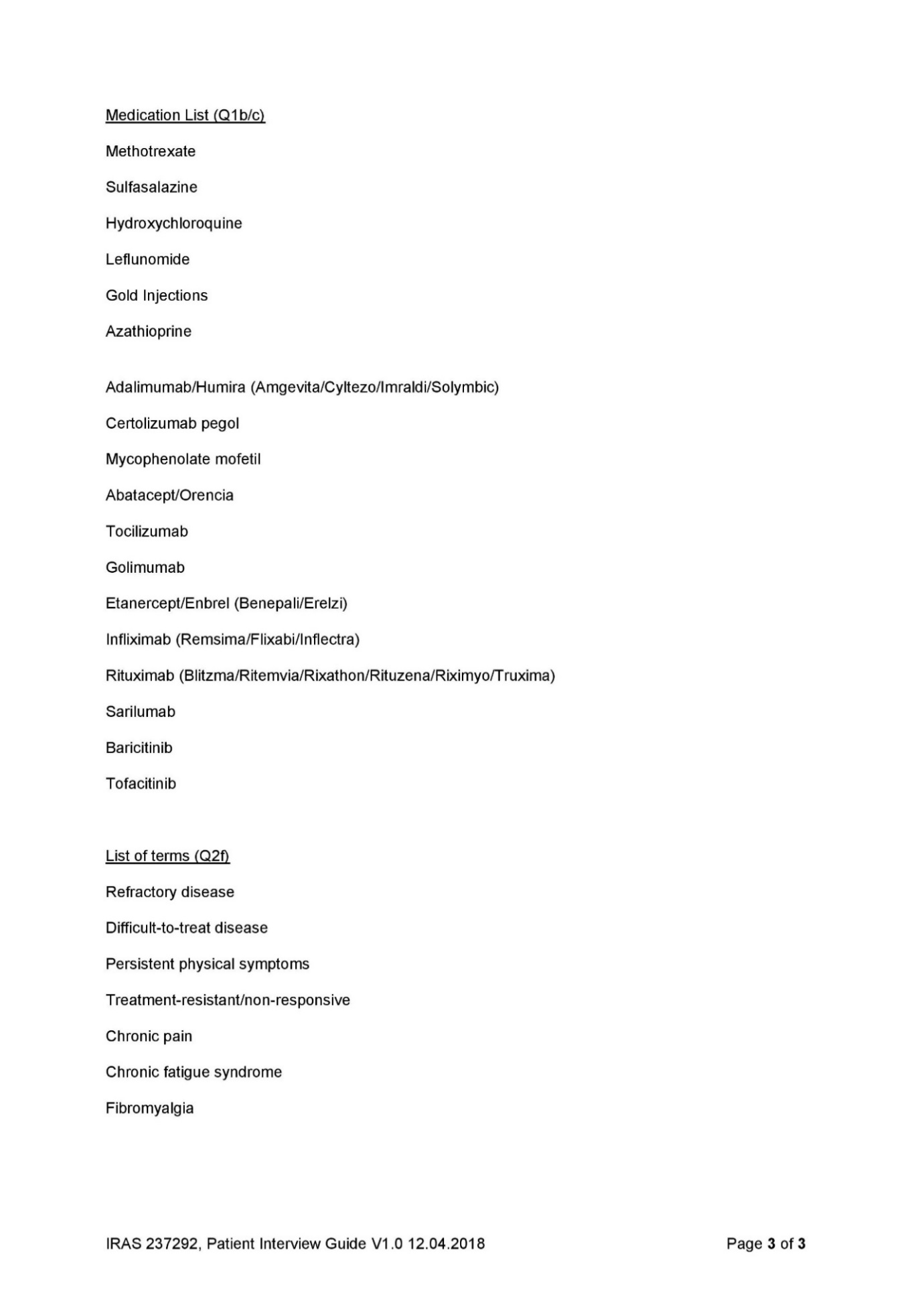


### Supplementary Information S1: B) HCP Interview Schedule


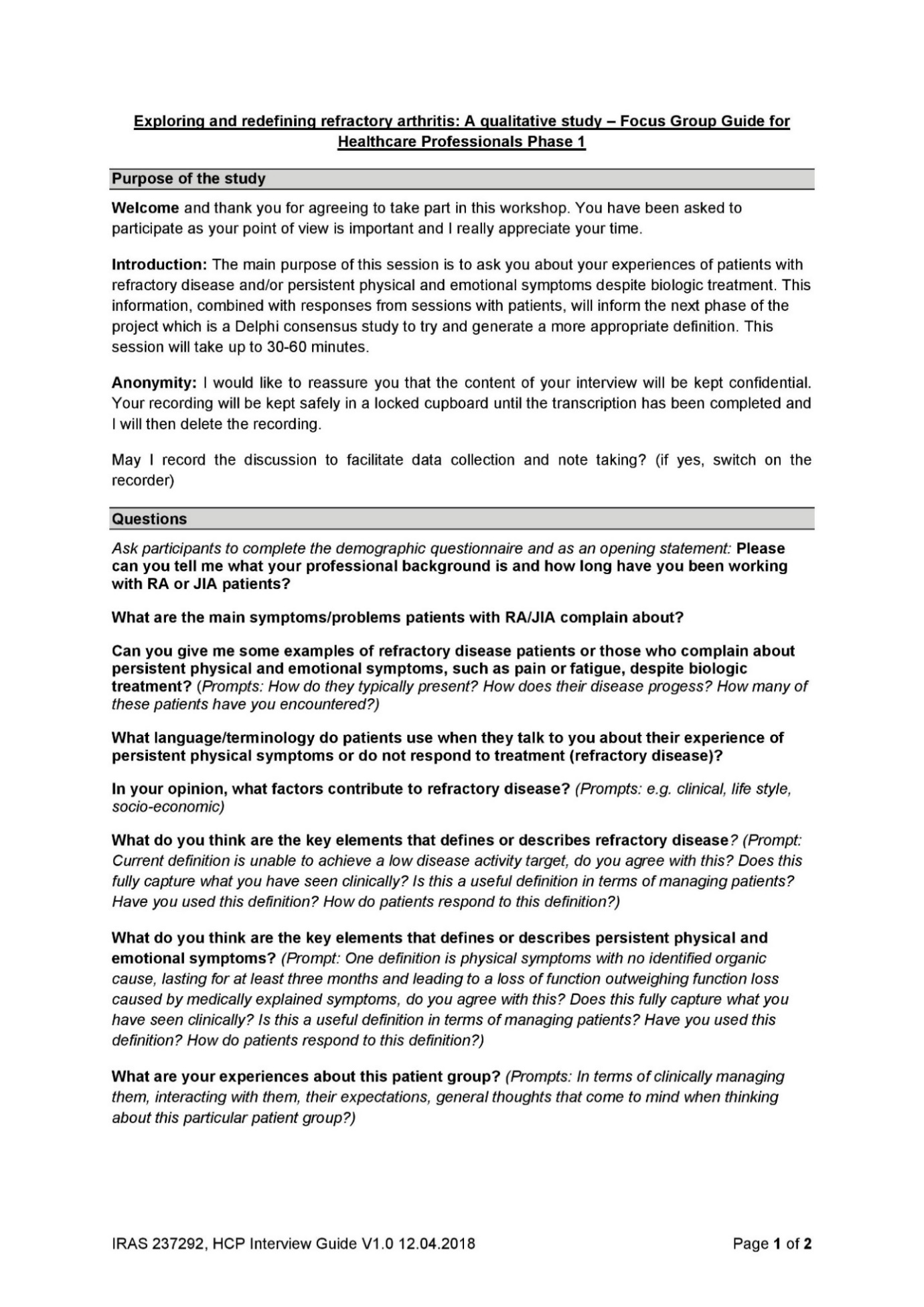


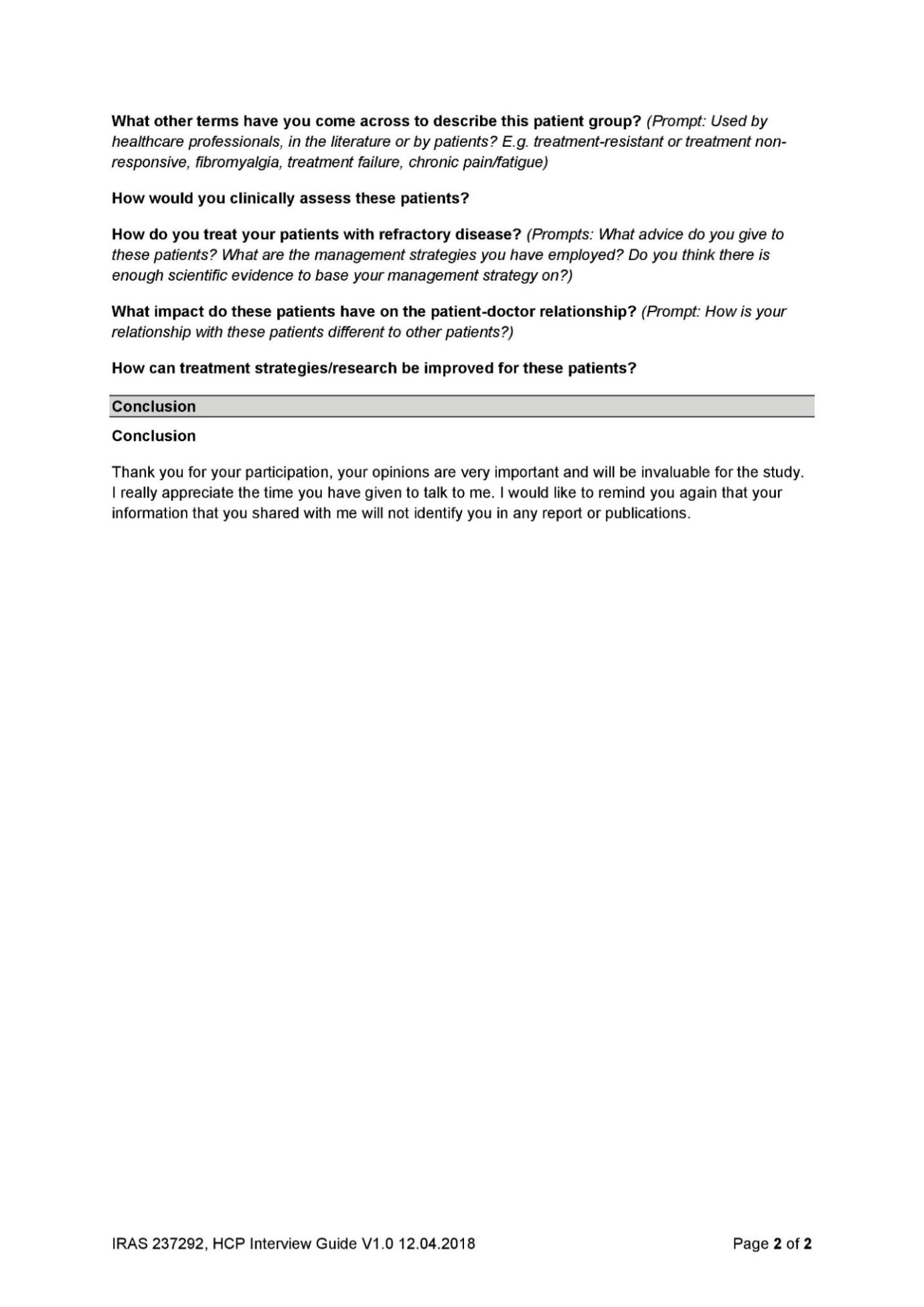

Supplement: Supplementary file 1 — Appendices S1–S5. [file BJHP-30-0-s001.zip › S2 - Interview Schedules.docx]
