# Supplementary material for: Characteristics of refractory disease and persistent symptoms in inflammatory arthritis: Qualitative framework analysis of interviews with patients and health care professionals
Source: Br J Health Psychol. 2025 Jan 8;30(1):e12780. doi: 10.1111/bjhp.12780 (PMC11707814; doi:10.1111/bjhp.12780)
Supplement: Supplementary file 1 — Appendices S1–S5. [file BJHP-30-0-s001.zip › S3 - Flowchart of participant recruitment process .docx]

**Supplementary Figure S3: A)** Flowchart of participant recruitment process for Patients and b) Healthcare Professionals


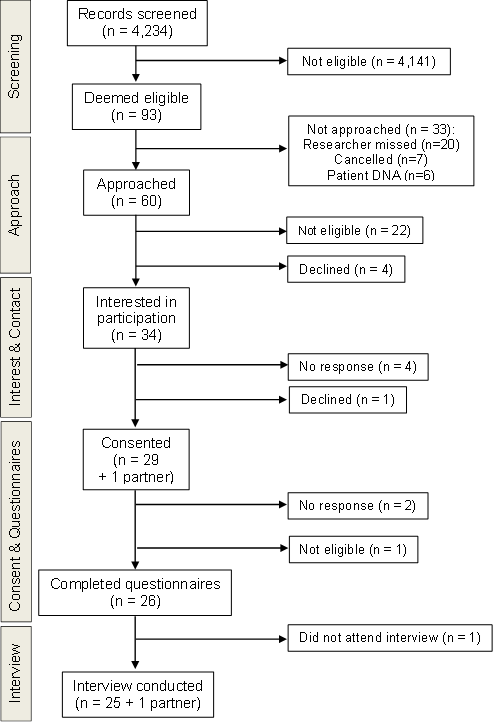


The patient recruitment process took place between August 2018-April 2019.

**Supplementary Figure S3: B)** Flowchart of participant recruitment process for Healthcare Professionals


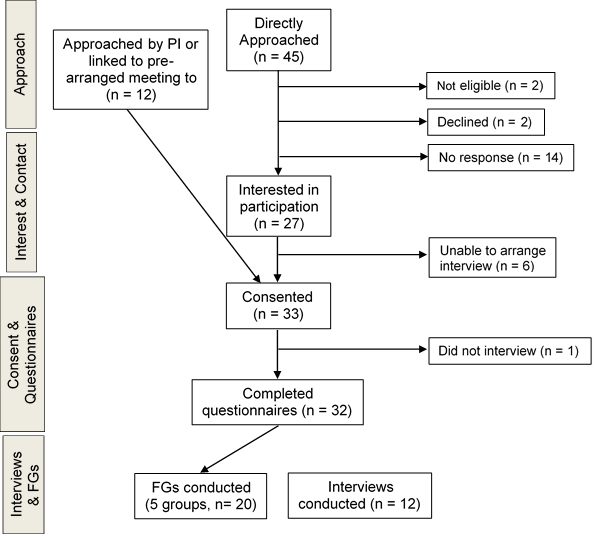


Recruitment process of HCPs took place between September 2018-January 2019.
