# Supplementary material for: Characteristics of refractory disease and persistent symptoms in inflammatory arthritis: Qualitative framework analysis of interviews with patients and health care professionals
Source: Br J Health Psychol. 2025 Jan 8;30(1):e12780. doi: 10.1111/bjhp.12780 (PMC11707814; doi:10.1111/bjhp.12780)
Supplement: Supplementary file 1 — Appendices S1–S5. [file BJHP-30-0-s001.zip › S4 - Framework Examples.docx]

### Supplementary Table S4: Example excerpts from Framework

|  | Participant | Independence/ Dependence on others | Support from family/partner – practically & emotionally | Life-limiting impact – restrictions physically, socially, vocationally |
| --- | --- | --- | --- | --- |
| RA - RD | PAT5A | *"I do most of the rough and tumble, like you know open, you know, all the lifting and opening jars and also lift and provide and, you know, she doesn’t have to do any like preparation, like dinner preparation all that, when I leave, I do it rather than she has to." [Partner]* | *“It’s not most of the housework, but you do the basics, not too much. If you say most of the housework, it’s not right because if you said most the housework that would be the hoovering, the cooking (laughter). She does the basics, just enough to, but I do the shopping.” [Partner]* | *"I had to change, but you know what I used to be, I used to be an auxiliary nurse, years ago and I, yeah, I had to give that [work] up then."* |
|  | PAT6A |  | *"it makes me feel awful because the family I have around me are older, my nan and grandad. Which again is quite, cos you should be looking after them, not them looking after you, but I get kind of a guilt that my family have to help me so much and I wanna be the one to help them. "* | *"Yeah, the activity with my daughter, obviously I've missed out on a lot. Just little things when she was younger, can't sit on the floor, you can't do certain [activities] that they want you to do, which can be quite frustrating (pause). Erm constantly having to cancel appointments cos you don’t feel well enough to be able to get out."* |
|  | PAT1B | *"it is very miserable not being able to do things, [chores/activities] you feel impotent. And, erm, people help you, which is kind, but it, that’s not really what you want, right. I mean, I know people do it with the best of intentions but that is not helpful, you want to be able to do it yourself, you know." "I still feel like I’m quite an independent person so I’m grateful for, for that [independence]."* |  | *"I would say [RA] has been life-limiting, without a doubt. Erm, yes, I was, I was quite a walker, I was quite, I would wander around and suddenly that was not available for me to do. So that was, I would say, erm, has been, yeah, significant. I've had to rethink, erm, my life. And I’ve had to absolutely include every decision I make, I have to include my illness. So whether that is, you know, can I get to work at a certain time. Can I, erm, can I actually physically get there. When I’m there are there adaptations. So I’ve worked for, so I worked for a government organisation for a long time because they do all that stuff for you. They’ll, you know, they have sort of good policies about time to get to work and adapting to those sorts of needs. So I would say I was pushed, not pushed, but I chose that path where there's a bit more of a nurturing responsibility rather than private sector."* |

|  | Participant | Systemic/flu-like feeling | Old age, co-morbidities or IA? | Challenges of being young with chronic illness |
| --- | --- | --- | --- | --- |
| PolyJIA - RD | PAT6C |  | *"Anything at the moment can make me feel really exhausted, even if I'm not doing anything too much, but with always having anaemia, it’s always been on and off, it’s a struggle of deciding where the fatigue’s coming from...So it’s difficult to decide which area that’s placed in because I’ve also struggled with some mental health issues as well, that can make me feel drained. So, again, [symptoms’] sort of a mix of all three things."* | *“Amount of things that I can do compared to people my age is extremely different…especially during a flare when you can't do anything, you can't go out, you can't go to lectures or write.” "if you were to speak to anyone in my age group, it’s always like you feel like an outsider (hmm) because of [PolyJIA] and the constant hospital appointments, the infusions, the tablets and everything else"* |
| PolyJIA - PPES | PAT9D |  | *"I'm diagnosed with headaches as well, migraine, so that affects me more than my joint problems when I do my uni work cos I can't concentrate, like I just want to go to sleep. So, yeah, I think my headaches, I'd rather have my joint problems than my headaches (laughs), cos my headaches are so bad."* | *"when I had it I think no one really knew back then [about PolyJIA] and some people, some teachers, when I had like days off and in the beginning when I had like days off and some other people used to get extra time, but I didn’t get extra time, so I was like upset, like they still don’t understand what I'm going through."* |
|  | PAT11D | *"I don’t get the kind of big body flare ups, like very swollen joints, but I would still say [PolyJIA] was active in that I will say: “I'm having a flare-up” like I will feel like I'm having a flare-up and it won't, but I won't necessarily get those, you know, big swellings, swelling and things like that, which other people might think of me as having a flare-up, if that makes sense (hmm), but I, erm, sometimes I’ll have specific joints that will hurt and then sometimes I also think I'm having a flare-up, but then it’s more like a systemic feeling; I know that it’s my joints, maybe if I've done like two long days at work, I've been on my feet, and then the next day I really feel that kind of, that heaviness in my joints and it will just feel erm, yeah, like I'm just systemically like heavy, like unwell, if that makes sense (hmm) and I’ll know that, it’s a very specific feeling and I’ll know that it’s like kind of the arthritis" "[symptoms are] almost that like flu-like feeling, like I just feel, it’s just a very systemic feeling and yeah."* | *"[pain] was in my sacroiliac joint, so I found that, again, the connotations of like back pain and like, “Oh God, you know, am I just getting older” like or, well, I thought at first it was cos I had a UTI cos it was a back pain there"* | *"I don’t want to be talking about what I'm gonna potentially have to do, like considerations for pregnancy when I was 15." "And yeah, just also like saying to people like, “Arthritis”, people are like, “What? You're not 80” or like those connotations and not wanting to feel like I'm 80 as well, like you know, like those days when I'm swollen and I'm sore and I think ugh, I shouldn’t feel like this at this age."* |

|  | Participant | Patient health beliefs and behaviours |
| --- | --- | --- |
| Adult - Rheumatology Registrar | HCP3B | *"I think [RD patients] come in at a very different place than someone who has a new diagnosis or is just beginning their journey in Rheumatology so that is often helpful cos you can talk through things and they’ve got a better understanding."* |
|  | HCP4B | *"And I sometimes think understanding as well. Like if people understand what, you know, why we’re giving them treatment and how it’s gonna help I think that [understanding] sometimes does help." "I think the patients that use the term ‘Fibromyalgia’ about themselves seem to have a better understanding, in that some of their pain isn’t going to be helped by upping their steroids or. I think they’re the ones that have accepted that there is another element to their ongoing symptoms."* |
|  | HCP5D |  |
|  | HCP6D | *"understanding. So I think if you understand what, what the problem with your arthritis is and you understand that the treatment will be immunosuppressive in nature and you understand how to take it and when to take it and when not to take it, then I think that will all definitely support. I think if, if you have patients who kind of for one reason or another: whether we’ve not explained it to them properly or they’ve not, not really paid attention to what we’ve said or don’t understand it. Taking medication the wrong way can be a problem and certainly, you know, I've seen instances where I thought I’d counselled someone perfectly for Methotrexate and then they’ve come back and their disease is still active and I’ll say: “What day do you take your Methotrexate on?” and they’ve said: “Oh, well, I've take [medication] on a Monday some days and some Tuesdays and Wednesdays some days.” but you’ve said take it once a week and so it’s a case of perhaps, you know, a lack of understanding in terms of medicine or a lack of understanding why they need the medicine for their treatment and in particular in something like Hydroxychloroquine, although it’s mild if you tell the patient this is not going to work immediately then sometimes it means they persist with it, whereas if you’ve just gone: “Take this, this will make your arthritis better.” And they go and take it for a week and go: “Ah, actually my arthritis is still bad.” Which, you know, would not be unexpected, then they may just decide to discontinue it."* |
| Adult - Clinical Nurse Specialist | HCP2A |  |
|  | HCP6C | *"sometimes when you’ve done like a disease activity score erm and it, you haven’t felt any active synovitis, [patients] are querying why you haven't felt it rather than the fact that it’s not there (Hmm). Erm, and basically wanting you to give them something to help even though you’re trying what you’ve got available"* |
|  | HCP2D | *"social media definitely plays a part in [patient knowledge] but also a lot of the stuff that’s come out now, was particularly with the cannabis oils and things like that where a lot of them are emailing me about that and are wanting to look into that side of things and: “I've heard of X, Y and Z in America and my friend’s mum has blablabla.” And you just, you try and tell them it’s not the same, so I think it’s how they view actually their specific type as well and not compared to what their gran had or what their mum had or their friend’s mum had or. So a lot of it’s education too and how much they understand about their conditions."* |
| Paed - Clinical Nurse Specialist | HCP6E | *"But there’s also some of those families, aren’t there, where the child, the whole dialogue of the consultation, are you in pain today, darling? Does it hurt? So they're reminded, whereas other parents probably don’t even notice and so it becomes, there’s no point complaining about it, even if you’ve got it but those families where you're constantly being asked are you in pain? You're gonna think well, perhaps I am then. It’s all suggested to you isn’t it?"* |
